# Mining gut microbiome oligopeptides by functional metaproteome display

**DOI:** 10.1038/srep34337

**Published:** 2016-10-05

**Authors:** Jonas Zantow, Sarah Just, Ilias Lagkouvardos, Sigrid Kisling, Stefan Dübel, Patricia Lepage, Thomas Clavel, Michael Hust

**Affiliations:** 1 Technische Universität Braunschweig, Institute of Biochemistry, Biotechnology and Bioinformatics – Department for Biotechnology, Germany; 2 Technische Universität München, ZIEL Institute for Food and Health, Freising, Germany; 3 Micalis Institute, INRA, AgroParisTech, Université Paris-Saclay, Jouy-en-Josas, France

## Abstract

Pathogen infections, autoimmune diseases, and chronic inflammatory disorders are associated with systemic antibody responses from the host immune system. Disease-specific antibodies can be important serum biomarkers, but the identification of antigens associated with specific immune reactions is challenging, in particular if complex communities of microorganisms are involved in the disease progression. Despite promising new diagnostic opportunities, the discovery of these serological markers becomes more difficult with increasing complexity of microbial communities. In the present work, we used a metagenomic M13 phage display approach to select immunogenic oligopeptides from the gut microbiome of transgenic mice suffering from chronic ileitis. We constructed three individual metaproteome phage display libraries with a library size of approximately 10^7^ clones each. Using serum antibodies, we selected and validated three oligopeptides that induced specific antibody responses in the mouse model. This proof-of-concept study provides the first successful application of functional metaproteome display for the study of protein-protein interactions and the discovery of potential disease biomarkers.

Biomarkers are indispensable tools for diagnostics, which can be measured in different body fluids and, thus, are important for the disease management in clinics. Due to the ease of collection, blood serum is the most convenient source for biomarker measurements. Moreover, it circulates through all body regions and tissues and carries immunoglobulins which can be indicative of specific diseases. Countless diagnostic ELISAs and other assays rely on the detection of one specific immunoglobulin. Technological advances such as protein microarrays, peptide microarrays, or Luminex multiplex assays also allow fast and cost-efficient measurement of complex patterns of antibody responses^1^,^2^,^3^,^4^. However, the identification of the antigens that trigger these specific systemic antibody responses is still difficult and limits the application of these technologies.

Classical proteomic studies to identify pathogen proteins for diagnostic applications rely on the cultivation of the pathogen followed by 2D-PAGE and immunoblot of the proteome using patient sera and protein identification by mass spectrometry (MS)^5^,^6^,^7^. However, proteome analysis after *in vitro* cultivation does not allow the identification of proteins only expressed during host-pathogen interactions. To identify immunogenic proteins that are specifically important in pathogenesis, the pathogen has to be cultivated in direct contact with the host to ensure gene expression patterns reflecting the true pathogenicity situation. However, an inherent problem is that the pathogen proteome will most likely be overwhelmed by that of the host^8^. Additionally, two other groups of potential biomarkers also usually fail to be identified by classical proteome analysis: Proteins with a molecular mass smaller than 10 kDa or weakly expressed proteins may not be identified by 2D-PAGE/MS^9^,^10^,^11^.

As an alternative, microarrays with several hundred spotted proteins have been used to identify immunogenic proteins from pathogens^12^,^13^,^14^, *E. coli*-derived biomarkers for inflammatory bowel disease^15^, and exposure related antibody responses to proteins from *Plasmodium falciparium*^16^. Even though microarrays allow the screening of several hundreds of target proteins at a time, the technology is laborious and expensive as each protein must be produced in a recombinant manner. Peptide microarrays can be produced with potentially millions of individual sequences^4^ allowing to cover entire proteomes, but they are still limited to only detect antibodies binding to short linear epitopes.

Serologic expression cloning (SEREX), a technology developed in the 1990 s, relies on the expression of cDNA libraries in *E. coli* using lytic phage vectors^17^. Plaques containing the recombinant proteins are transferred to membranes and stained with patient sera. Immunogenic proteins are identified by DNA sequencing of corresponding clones. The SEREX technology was initially developed for serologic analysis of tumor cDNA expression libraries^18^, but was also used to screen expression libraries from metagenomic DNA^19^. However, handling of lytic phage, maintenance of the phage library, and laborious screening procedures are major drawbacks of this technology.

Instead of using phage for cytoplasmic expression libraries, heterologous proteins can be displayed on the surface of lytic phage (e.g. T7 phage^20^) or non-lytic phage particles^21^. In a subsequent panning procedure using a protein interaction partner immobilized on a solid surface, the phage particles displaying proteins which are specifically bound by the immobilized interaction partner are enriched at the solid surface.

Phage display using non-lytic filamentous M13 phage is enabled by fusing the heterologous protein to a phage coat protein, mostly minor coat protein III (pIII) and is mostly used for the selection of antibodies today^22^,^23^,^24^. However, the ability of peptide phage libraries to be enriched for epitopes of monoclonal antibodies has long been demonstrated^25^. As phage particles contain the genetic information of the displayed proteins, genotype and phenotype are linked. Hence, the technology facilitates not only enrichment but also the simple identification of protein interaction partners by DNA sequencing, e.g. immunogenic proteins which induced systemic antibody responses. Phage display can be used to display oligopeptides and full protein domains derived from a natural source such as genomic DNA or cDNA. However, when randomly fragmented DNA is cloned in a non-directional manner, only one out of eighteen clones result in open reading frames (ORF) and the enrichment of ORFs is a crucial step to improve the library quality. ORF enrichment in phage display libraries was accomplished by several different strategies, such as cloning the DNA fragments upstream of a selection marker like β-lactamase. Only fragments cloned in-frame with the β-lactamase gene and do not hamper correct folding allow the expression of functional β-lactamase and confer ampicillin resistance to the corresponding *E. coli* clone cultivated on selective media. Enriched ORFs are subsequently subcloned into a phage display vector^26^,^27^ or the β-lactamase gene is removed by CRE-mediated recombination^28^. In a T7 phage display approach to identify Calpain substrates, fragmented DNA was cloned upstream of a biotinylation-tag. This way, only ORF fragments could result in tagged and biotinylated gene products, characterizing a procedure similar to using antibiotics for selection^29^. Furthermore, immobilizing produced phage on a surface using streptavidin allowed an ORF selection and the identification of protease substrates by Calpain-mediated release from the surface.

Infectivity of the lytic M13 phage is mediated by pIII whereas each phage particle has up to five pIII copies. The N-terminal domain can be divided in two subdomains (N1 and N2) that mediate infection by binding to the host cell’s F pilus, whereas the C-terminal domain (CT) anchors the protein in the phage capsid. These three regions are separated by glycine and serine rich linkers. Introducing trypsin sites into the linker regions renders pIII sensitive to proteolytic cleavage and decreases infectivity after trypsin treatment. Cloning randomly fragmented DNA upstream of a trypsin resistant pIII gene in a phagemid vector and using a trypsin sensitive helperphage for phage packaging allows ORF enrichment of genomic libraries after trypsin treatment and infection of *E. coli* host cells^30^.

Using another special helper phage which has pIII in its capsid and is infective but has a genomic deletion of the pIII encoding gene *gIII* (“Hyperphage”^31^,^32^), ORFs can be enriched without further cloning steps or protease treatment (ORFeome phage display^33^,^34^). Therefore, randomly fragmented DNA is cloned upstream of the pIII gene on a phagemid vector. After “Hyperphage” co-infection, infective phage particles are only produced if the inserted DNA sequence is in-frame with the pIII gene and does not contain any stop codon as the fusion protein is the only pIII source to render the phage particles infective. Consequently, ORFs are strongly enriched during “Hyperphage” packaging.

The ORFeome phage display technology was recently used to identify novel potential biomarker or vaccine candidates from genomic libraries of different *Mycoplasma* species^35^,^36^, *Salmonella* Typhimurium^37^, *Neisseria gonorrhoeae*^38^, and from a tick salivary gland cDNA library^39^. These studies used functional proteomic display of oligopeptides from single species, but since the ORFeome phage display technology is independent of cultivation, it is in principle usable for the study of any ORF containing DNA mixture. Complex microbial communities such as the intestinal microbiota could be analyzed by functional screening of metaproteome phage display libraries when using metagenomic DNA preparations.

Crohn’s disease (CD) and ulcerative colitis (UC) are idiopathic relapsing chronic inflammatory disorders of the gut commonly referred to as inflammatory bowel disease (IBD). Genetic predisposition markers comprise mutations in genes associated with microbial sensing^40^,^41^,^42^. Moreover, shifts in microbiota composition are associated with IBD^43^,^44^, highlighting the crucial role of the microbiome in IBD pathogenesis. Hence, IBD is closely correlated to aberrant immune responses towards the gut microbiota in a genetically susceptible host. Humoral immune responses in IBD patients include autoantibodies like anti-neutrophil cytoplasmic antibodies (ANCA) and increased immunoglobulin levels against microbial antigens such as cell-wall components of yeast (anti-*Saccharomyces cerevisiae* antibodies, ASCA), bacterial flagellin (anti-Cbir1), *Escherichia coli* outer membrane porin C (anti-ompC), and *Pseudomonas fluorescens* protein I-2 (anti-I2). For IBD diagnostics, ASCA have the best combined sensitivity (31–45%) and specificity (90–100%) (reviewed in ref. 45). However, due to the low prevalence of already known IBD-associated antibody responses, there is an urgent need for additional serologic markers that may support IBD diagnostics and give further insights into host-microbiome interactions.

In order to study molecular mechanisms underlying IBD, numerous mouse models have been established (for review see ref. 46). Transgenic TNF^ΔARE/+^ mice are characterized by elevated TNF levels and spontaneous development of CD-like ileitis^47^. Ileal inflammation in this model was recently shown to be triggered by the gut microbiota, as germ-free mice did not develop inflammation and mice deficient in MyD88 (an adaptor protein essential for innate recognition of gut microbes) showed attenuated signs of inflammation^48^,^49^. Moreover, shifts in the microbiota associated with inflammation were able to induce ileal inflammation when transferred to germ-free animals^48^. Hence, involvement of both TNF and the gut microbiota are shared features between TNF^ΔARE/+^ mice and human CD, suggesting mechanistic similarities in the pathophysiology, making this mouse model well-suited to study host-microbiome interactions.

Mining the gut microbiome for immunogenic bacterial proteins capable of inducing a systemic antibody response in the host may reveal novel biomarker candidates for IBD diagnostics. In the present work, we performed a proof-of-concept study on the development and use of functional metaproteome display for biomarker discovery.

## Results

### Construction of metaproteome phage display libraries

**Figure 1:**
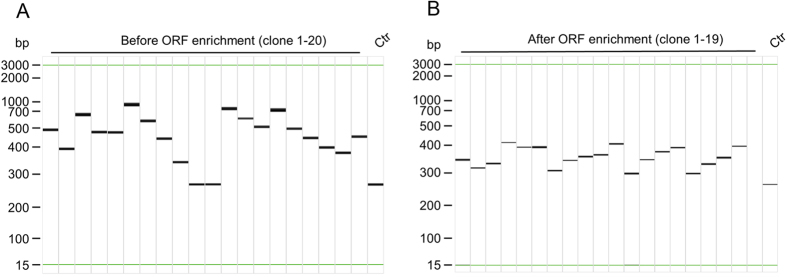
Representative colony PCR of random clones of a metaproteome library before and after ORF enrichment. (**A**) Lane 1–20: randomly analyzed clones before ORF enrichment. (**B**) Lane 1–19: randomly analyzed clones after ORF enrichment. Ctr: empty pHORF3 vector backbone. Colony PCR was analyzed by capillary electrophoresis and results are displayed as virtual gel image. Insert rates and mean insert sizes were estimated by colony PCR of 20 clones per transformation (n = 4) of each library and after Hyperphage packaging of each library.

Three individual metaproteome phage display libraries were constructed from metagenomic DNA isolated from the caecum of three 8-week-old TNF^ΔARE/+^ mice (illustrated workflow in Figure S1A). Based on transformation rates, the initial library diversities were 6 × 10^7^, 2 × 10^7^ and 3 × 10^7^ independent clones. Insert rates were above 85% for all three libraries with mean insert sizes of 284 to 354 bp as analyzed by colony PCR (Fig. 1A). Library quality was further enhanced by the enrichment of ORFs and the corresponding oligopeptides were displayed on phage particles (illustrated in Figure S1B). ORF enrichment also removed empty vector backbones (100% insert rate) and mean insert sizes were decreased to 92 to 117 bp after ORF enrichment (Fig. 1B).

### Gut microbiota sequencing

**Figure 2:**
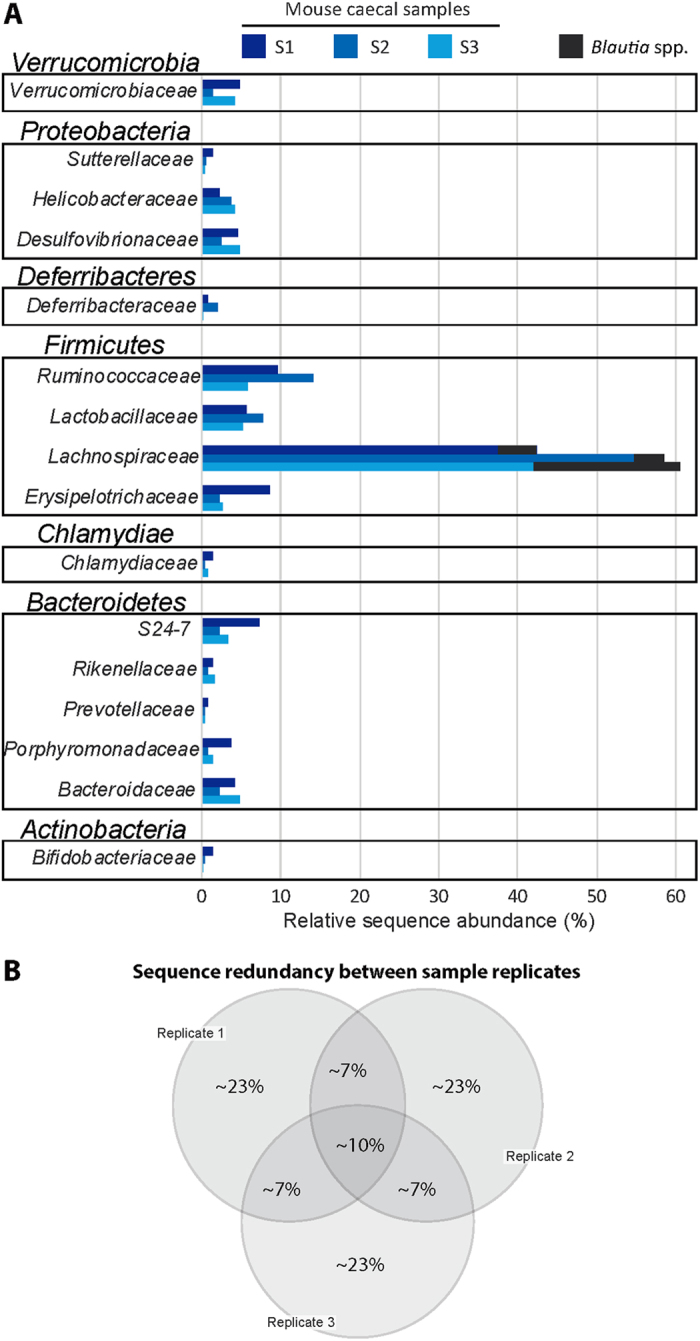
Gut microbiota and phage oligonucleotide sequencing. (**A**) Taxonomic composition of the three donor mice at the bacterial family level as per 16S rRNA gene analysis. Reads were classified using SILVA^70^ and families were ranked by phyla. Relative abundances refer to the proportion of sequences belonging to the given taxa considering a mean number of sequences per sample of approximately 17,000. (**B**) Venn’s diagram showing the percentage of oligonucleotides that are shared between or unique to the replicate libraries for any given mouse caecal sample. The percentages refer to a total number of approximately 265,000 unique oligonucleotide reads.

To assess gut bacterial diversity and composition in the three mice used to construct the phage libraries, DNA isolated from caecal contents was analyzed by high-throughput 16S rRNA amplicon analysis. The operational taxonomic unit (OTU) table summarizing sequence counts of each molecular species per sample together with the corresponding OTU sequences are provided in File S1 and S2, respectively. Samples were characterized by low alpha-diversity, i.e. an average species richness of 56 and a Shannon effective species count^50^ of 24. The taxonomic composition per sample (Fig. 2A) was in line with data from the literature. The dataset was characterized by marked inter-individual differences (e.g. OTU-1 was abundant in sample S2 and S3, but not in S1). However, more than 50% of the reads were classified as member of the phylum *Firmicutes* in all samples, with relative abundance of OTUs corresponding to members of the genus *Blautia* (see candidate peptides below) ranging between 4 and 18%.

### Phage oligonucleotide diversity and size distribution

In order to obtain an overview of the starting oligonucleotide diversity prior to panning, triplicate phage libraries of each of the three donor mice were sequenced. An average of 193,500 total reads (about 176,000 after merging and excluding reads with more than 0.1% expected error rate) and 122,000 unique reads were obtained per mouse per replicate. The mean sequence length of each library was 84.8 ± 1.9 nt, 65.8 ± 4.3 nt and 71.7 ± 5.7 nt with maximum insert lengths of 499 nt, 448 nt and 492 nt, respectively (size limit due to sequencing specifications was approximately 500 nucleotides).

Approximately 30% of the reads (54,000 out of 176,000, represented on average by 30,000 unique peptides) corresponded to redundant peptides (i.e., those with more than one copy; median copy number was 2, showing that most of the redundant peptides had low copy numbers; maximum copy number ranged between 44 and 163, depending on the library). The 20 most abundant clones represented less than 0.5% of total reads. Across the reads obtained from the triplicate library preparations of the same sample, only 10% of the sequences were shared between all three replicates and another 7% between each of the replicate pairs (Fig. 2B). These findings suggest that the total diversity of oligonucleotides existing in the libraries was not covered by either the individual library preparation or more likely the number of sequencing reads, and exceeded the 265,000 detected sequences per library. Most of all, these data show that the constructed library displayed a high degree of diversity as unique peptides dominated the libraries, demonstrating their suitability as starting materials for metaproteomic biomarker candidate selection.

Detailed NGS analysis of one replicate of ORF enriched library 1 (Table S1) revealed a size distribution ranging from 8 to 476 nt. Random fragmentation of DNA for library construction results in DNA fragments with sizes of 3n, 3n + 1 and 3n + 2 nucleotides. Sequences with a length of 3n + 1 were enriched over sequences with 3n + 2 and 3n nucleotides (70.3% vs. 27.3% vs. 2.4% of unique reads) (Figure S2). Due to the vector design (Figure S3), only inserts with 3n + 1 nucleotides constitute ORFs without a frameshift in the pIII gene. Only 3.4% of inserts with 3n + 1 nt contained stop codons (vs. 2.7% and 43.0% for inserts with 3n + 2 and 3n nt, respectively). Notably, out-of-frame inserts with 3n + 2 nucleotides were less efficiently removed during ORF enrichment than inserts with 3n nucleotides and contained significantly less stop codons. A fraction of 17.4% of inserts with 3n + 1 nt and one or more stop codons could potentially be rescued as they also contained a second start codon in the correct frame to translate into a pIII fusion protein. Moreover, alternative start codons for the potential translation of a pIII fusion protein were detected in about 30% (30.0% and 28.4% of inserts with 3n + 2 and 3n nt, respectively) of the out-of-frame inserts. Taking alternative start codons into account, a total of 77.2% of analyzed sequences had a potential gene product comprising an oligopeptide:pIII fusion protein. Hence, these sequencing data demonstrate the efficiency of the ORF enrichment step.

### Selection of immunogenic oligopeptides from metaproteome phage display libraries

To select microbiota-derived immunogenic oligopeptides from the metaproteome phage display libraries, three independent pannings using immobilized serum antibodies (IgA, IgG and IgM in combination) were performed. The complete workflow is illustrated in Figure S1C. Paired samples (i.e. one individual library constructed from a given mouse gut content and the corresponding serum sample) were used. After three consecutive rounds of panning (output of selection rounds is given in Figure S4), 92 clones from each panning were screened by monoclonal phage ELISA aiming to identify specific antibody response of the corresponding sera towards the displayed oligopeptides. In total, 16 individual clones reactive with the designated mouse serum were selected (Table 1). Some selected clones were found in multiple copies among the 276 screened clones. In particular, clone JOZ156-A6 was dominantly enriched during the panning procedure, but was later found to be a universal immunoglobulin binding protein (data not shown). The selected fragments ranged from 31 to 328 bp in size. Protein homologies (blastp) were only found for clones JOZ157-E1 and JOZ158-E11. Clones JOZ156-A6, JOZ156-A8, JOZ156-F7, and JOZ157-C10, were annotated as predicted proteins from *Blautia* species.

### Validation of immunogenicity by immunoblot

**Figure 3:**
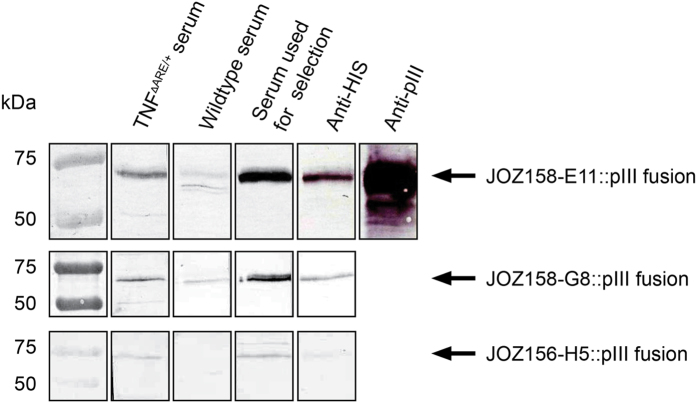
Immunoblot of oligopeptide phage particles stained with serum. Oligopeptide:pIII fusion protein was stained with serum pools (1:200 dilution) from TNF^ΔARE/+^ and wildtype mice. Bound antibodies were detected with an anti-mouse (IgA, IgG, IgM) secondary antibody. Control staining of mouse anti-hexahistidine and exemplary mouse anti-pIII (JOZ158-E11) staining was included to identify the pIII fusion protein.

Blotted proteins of the selected oligopeptide phage particles were incubated with pools of each 15 TNF^ΔARE/+^ and 19 wt C57BL/6N mouse sera at the age of 18 weeks. All TNF^ΔARE/+^ mice were inflamed in the distal ileum and proximal colon, as confirmed by histological scoring (3.9 ± 1.3 vs. 0.03 ± 0.08 (distal ileum) and 4.3 ± 1.7 vs. 0.05 ± 0.13 (proximal colon)). Detection of bound serum antibodies resulted in a stronger staining of the oligopeptide::pIII fusion protein for three of the 16 candidate oligopeptide phage clones (Fig. 3). These peptides (JOZ156-H5, JOZ158-E11, and JOZ158-G8) were considered to be the most promising candidates for detection of specific circulating antibodies in TNF^ΔARE/+^ mice and were synthesized as biotinylated peptides.

### Validation of TNF^ΔARE/+^ specific immune responses by ELISA

**Figure 4:**
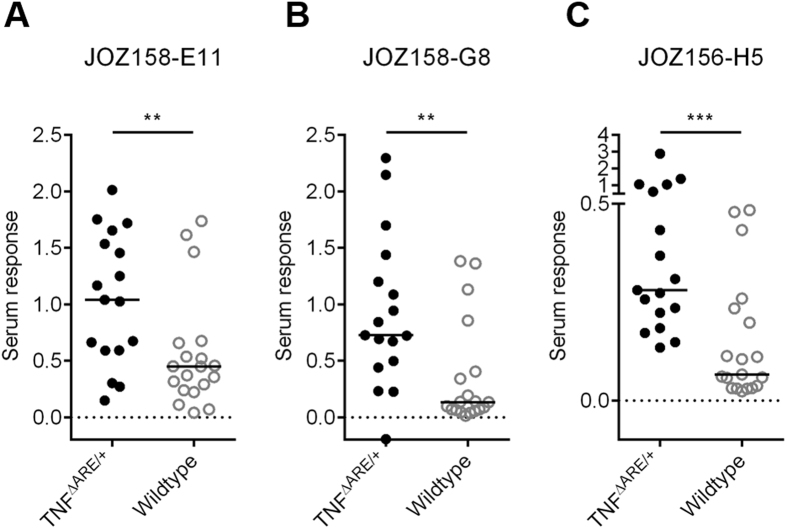
Specific serum reactivity against selected peptides in ELISA. Reactivity of TNF^ΔARE/+^ and wildtype sera with (**A**) JOZ158-E11 oligopeptide (**B**) JOZ158-G8 oligopeptide (**C**) JOZ156-H5 oligopeptide. The median cohort reactivity is indicated by a black line. Selected peptides were immobilized by streptavidin and bound serum antibodies from single sera (1:100 dilution) were detected with an anti-mouse (IgA, IgG, IgM) antibody. (Non-parametric Mann-Whitney test; ** = p < 0.01; *** = p < 0.001)).

Even though immunogenicity was validated using oligopeptide phage and serum pools, further characterization was performed using synthetic peptides and individual sera of the TNF^ΔARE/+^ and control cohorts. Peptides were immobilized via streptavidin and incubated with the individual sera. Bound anti-peptide serum antibodies were detected by ELISA using a secondary anti-mouse antibody. Some sera of mostly the TNF^ΔARE/+^ cohort reacted with streptavidin alone and competition with streptavidin in solution did not decrease the background binding of streptavidin. Therefore, a background subtraction step was implemented to exclude false positive reactive sera. The measured antibody responses against all three peptides were significantly higher in the TNF^ΔARE/+^ than wildtype mice, despite marked inter-individual differences (Fig. 4). Compared to peptide JOZ156-H5, antibody responses against the peptides JOZ158-E11 and JOZ158-G8 were generally stronger and reactivity of some control sera was increased. Nevertheless, the serum reactivity of the TNF^ΔARE/+^ cohort against both peptides (JOZ158-E11 and JOZ158-G8) was significantly increased compared to the control sera. Consequently, all three isolated peptides can be considered as biomarker candidates for the TNF^ΔARE/+^ mouse model.

The sequence JOZ158-E11 derived from a protein of *Blautia* sp. with predicted function of a phosphoribosyl aminoimidazole succinocarbox amide synthase (SAICAR synthase) based on the identification of the conserved domain and the similarity to the already annotated protein (Table S2). The identification of the closest homolog was performed with a blastp search over the nr protein database in NCBI.

### Time-dependent occurrence of the biomarker candidates

**Figure 5:**
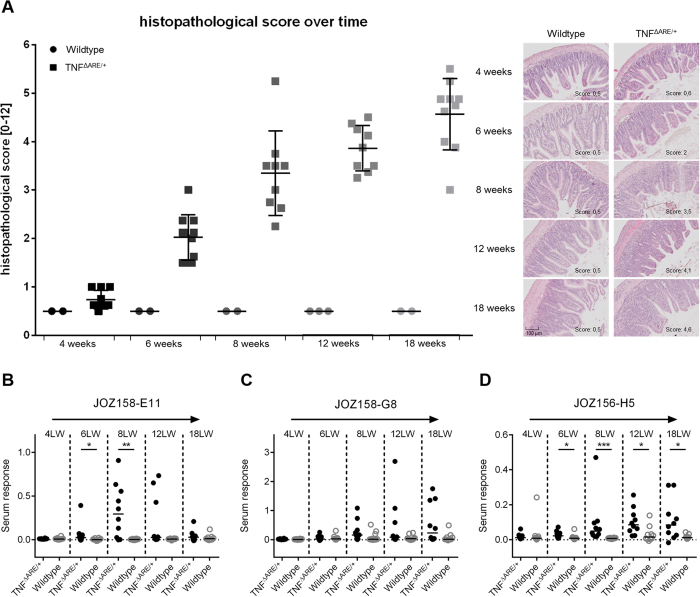
Time course of serum reactivity. Antibody response of mice sacrificed at life week 4, 6, 8, 12 and 18. (**A**) Histologic scoring of ileal inflammation (only 2 out of 10 wildtype samples were scored); serum reactivity with (**B**) JOZ158-E11 oligopeptide (**C**) JOZ158-G8 oligopeptide (**D**) JOZ156-H5 oligopeptide. The median cohort reactivity is indicated by a black line. Selected peptides were immobilized by streptavidin and bound serum antibodies from single sera (1:100 dilution) were detected with an anti-mouse (IgA, IgG, IgM) antibody. (Non-parametric Mann-Whitney test; * = p < 0.05; ** = p < 0.01; ***p < 0.001).

TNF^ΔARE/+^ mice are known to develop intestinal inflammation gradually from the age of 4–6 weeks on with plateauing around 12 weeks of age^47^,^51^. To test whether the systemic antibody response towards the three identified gut microbiome-derived peptides correlated with age and inflammatory state, serum reactivity was monitored using ten mice of each genotype sacrificed at week 4, 6, 8, 12, and 18 of age. Intestinal inflammation was assessed by histopathological scoring and anti-JOZ156-H5, anti-JOZ158-E11, and anti-JOZ158-G8 serum antibody titers were measured by ELISA (Fig. 5). Intestinal inflammation was observed by week 6 and the inflammatory state increased with older age of the TNF^ΔARE/+^ mice, whereas no inflammation was observed in wildtype controls (Fig. 5A). Inter-individual differences in serum response were observed for all three analyzed oligopeptides. Levels of anti-JOZ158-E11 antibodies were relatively high in 8-week-old TNF^ΔARE/+^ mice, but were not further increased thereafter and were not abundant in the serum of 18-week-old mice (Fig. 5B). Anti-JOZ158-G8 antibodies were only present in a fraction of TNF^ΔARE/+^ samples with only a trend towards increased response from week 8 on (p = 0.079 and 0.078 for 12 weeks and 18 weeks, respectively) (Fig. 5C). Serum levels of anti-JOZ156-H5 antibodies were increased in 4-, 6-, 8-, 12- and 18-week-old TNF^ΔARE/+^ mice when compared to the matched wildtype controls (Fig. 5D).

## Discussion

The present work identified three novel immunogenic oligopeptides from gut microbiota-derived metagenomes in a mouse model of Crohn’s disease and thus demonstrates that ORFeome phage display is suitable to analyze metaproteomes. To date, ORFeome phage display technology was only used to identify immunogenic proteins from single organisms^35^,^36^,^37^,^39^. Ciric *et al*. 2014 used a similar technology for the enrichment for DNA inserts that contained an endogenous signal peptide and analyzed this secretome enriched library by next generation sequencing, but this approach did not include any functional screening^52^,^53^. Here, we present an approach that allows to identify immunogenic proteins from ORF-enriched phage display libraries based on specific protein interactions including functional selection from proteins of a metaproteome independent of cultivation, expression level or antigen size.

Compared to phage display libraries covering genomes of one species, metagenomic approaches require larger libraries. Estimating the necessary library diversity to cover the whole metagenome remains difficult as the microbiome composition and relative abundance of the different species is not completely known and is highly individual-specific. The libraries constructed in the present work contained more than 10^7^ individual clones. In a previous study, serological expression cloning (SEREX) of randomly fragmented metagenomic DNA from the caecum of C3H/HeJ Bir mice and screening of 6 × 10^5^ pfu of the lambda expression libraries was sufficient to identify bacterial flagellin as immunogenic protein^19^. Moreover, SEREX relies on non-directional cloning of randomly fragmented DNA, so only a small fraction (typically less than 6%) of inserts usually results in open reading frames. Employing non-lytic M13 phage for the ORFeome phage display technology as in the present study not only allows the screening by monoclonal ELISA but also an ORF enrichment prior to selection. Hence, ORFeome phage display libraries contain a much lower number of junk fragments which can hamper functional selection by their stickiness. Although our libraries contained more than 10^7^ clones and up to 20 Gbp, they presumably still not covered the entire metagenome. The human gut microbiome harbors several hundred different bacterial species^54^ with more than 10 million genes^55^,^56^. Genes from species with low abundance may be underrepresented. Additionally, the metagenomic DNA used for library construction may have also contained DNA of other microorganisms such as fungi and protozoa as well as viruses, host, and food-derived DNA.

Nevertheless, the constructed phage libraries had a substantial degree of diversity that allowed their use as starting materials for peptide selection. Moreover, the ability to perform culture-independent proteomic studies is one of the superior characteristics of the technology, clearly demonstrated as only 56% of 16S rRNA reads detected by sequencing have been related to cultured bacteria at the genus level^57^.

In the present work, the ORF-enriched libraries were analyzed for the first time by NGS analysis and revealed that few clones were overrepresented in the libraries but the majority of sequences were equally distributed. In another NGS study analyzing a commercial peptide phage library (Ph.D.™-12) after amplification, the authors acquired 2 × 10^7^ reads to analyze a library of 10^6^ unique sequences^58^. These are about 50-times more reads compared to the present study suggesting that we did not cover the full library diversity especially considering that only 10% of sequences were shared between NGS analysis of replicate library preparations. Nevertheless, we were able to gain insights into the size distribution and relative abundance of individual clones in the libraries. In the previous study with the commercial Ph.D.™-12 peptide phage library, the authors reported a strong bias towards 150 clones that dominated 20% of the library (with 20 clones accounting for 8%)^58^. We expected an even stronger bias in the present study due to the different insert sizes ranging from 8 to 476 bp having different impact on cell growth. However, the 20 most abundant clones accounted for less than 0.5% of total reads, indicating the lack of a respective bias in our libraries. The size distribution of nucleotide inserts revealed a strong enrichment (70.3% of total sequences) of clones with inserts of 3n + 1 nucleotides and only in a minor fraction (3.4% of sequences with 3n + 1 nt) remained stop codons. The remaining 29.7% of the packaged ORFeome phage display library had 3n and 3n + 2 nt inserts, which were out-of-frame with the pIII gene and in theory they should not result in infective phage particles. However, about 30% of each of these two types of out-of-frame inserts contained alternative start codons as potential translational start points for a pIII fusion protein which renders the phage clone infective. This phenomenon was described in a previous study when oligopeptide phage clones with out-of-frame inserts and alternative start codons coding for an immunogenic protein were selected and validated^38^. From insert size distribution in combination with detailed stop codon analysis of the obtained NGS data we concluded that in total more than 77% of the library contained potential ORFs demonstrating the efficacy of the ORF enrichment using the “Hyperphage” technology. The degree of enrichment is in line with previously reported ORF enrichment using the ORFeome phage display technology^34^,^38^. Further, functionally displayed oligopeptides derived from inserts with frameshifts were described before^34^,^59^,^60^ and were proposed to result from RNA secondary structures or a selection pressure against oligopeptides which may be toxic for the *E. coli* host. Therefore, an enrichment for 100% ORFs may not be possible at all.

We selected two immunogenic oligopeptides of 20 and 56 amino acids that could not be identified by BLAST analysis. The third selected oligopeptide comprised 40 amino acids of a phosphoribosylaminoimidazole-succinocarboxamide synthase (SAICAR synthase) of *Blautia* sp. and is very similar to SAICAR synthases from other highly abundant members of the mammalian gut (e.g. *Dorea* sp. and *Clostridium* sp.). SAICAR synthase is an enzyme involved in the *de novo* biosynthesis of purine nucleotides^61^ and essential for all living organisms. Due to structural and functional differences between microbial and vertebrate SAICAR synthases, selective inhibitors have been proposed as antimicrobial agents^62^ and structural differences may confer immunogenicity to bacterial SAICAR synthase. As intracellular protein, SAICAR synthase may have been exposed to the mouse immune system after bacterial cell lysis. In a previous study using the ORFeome phage display technology, the selection procedure allowed the identification of intracellular proteins from *Neisseria meningitidis* that have been described to be immunogenic before^38^.

Except from SAICAR synthase, protein function and genetic origin of the selected clones remain unknown. The fact that BLAST analysis of all 16 initially selected clones did not return any annotation from the NCBI nt/nr database with high homology on nucleotide level indicates that the gut microbiome may be more diverse than assumed and motivates to look for more so far unknown species in the mammalian gut^63^.

In conclusion, ORFeome phage display technology has demonstrated promising features for the identification of novel biomarkers in IBD and other microbiome-related diseases. Libraries may be constructed from any type of genomic or metagenomic DNA. Thus, ORFeome phage display may also facilitate the use of environmental metagenomes to identify protein interaction partners of microbial communities that may be of further interest for biotechnological or industrial processes.

## Methods

### Mouse experiments

Animal use was approved by ‘Landratsamt Freising’ (animal welfare authorization no. 32–568), following the guidelines of the ‘Deutsches Tierschutzgesetz’ (German Animal Welfare Act) and the ‘Deutsche Tierschutz-Versuchstierverordnung’ (German Animal Welfare of Experimental Animals Regulation) under supervision of a veterinarian and an animal welfare officer. Mice were sacrificed for scientific purpose only and were not included in any specific treatment protocols. Heterozygous TNF^ΔARE/+^ 47 and wildtype mice on a C57BL/6N genetic background were housed in the mouse facility at the Life Sciences faculty of the Technische Universität München under conventional conditions with a 12 h light/dark cycle at 24–26 °C. All mice were fed a standard diet (R/M-H, Ssniff, Soest, Germany) *ad libitum* and were sacrificed by CO_2_ inhalation at the indicated age (4 to 18 weeks).

### Histological scoring

Formalin fixed and paraffin embedded tissue sections of the distal ileum and proximal colon from TNF^ΔARE/+^ and WT mice were stained with hematoxilin and eosin (H&E) and scored in a blinded manner by assessing lamina propria mononuclear cell infiltration, crypt hyperplasia, goblet cell depletion and architectural distortion. Scoring resulted in a score from 0 (non-inflamed) to 12 (highly inflamed) per section as previously described^64^.

### Isolation of metagenomic DNA

Frozen caecal samples were mixed with 600 μl DNA stabilization solution (STRATEC biomedical), 400 μl phenol:chloroform:isoamyl alcohol (25:24:1; Sigma-Aldrich) and 500 mg autoclaved zirconia/silica beads (0.1 mm; BioSpec). Cells were disrupted by mechanical lysis using a FastPrep®-24 (3 × 30 sec at maximum speed) (MP Biomedicals). Samples were then heat-treated (95 °C, 5 min) and centrifuged (16,000× g/5 min/4 °C). Supernatants were treated with RNAse (1 μg/μL) for 30 min at 37 °C. Metagenomic DNA was purified using gDNA columns (Macherey-Nagel) following the manufacturer’s instructions. Concentrations and purity were measured using NanoDrop® (Thermo Scientific) and samples were stored at −20 °C.

### Construction of metaproteome phage display libraries

Metagenomic DNA (100 ng) was amplified by linear unbiased multiple displacement amplification according to the manufacturer’s instructions (illustra Ready-To-Go GenomiPhi V3 DNA Amplification Kit, GE Healthcare). The amplified DNA was diluted in 2 mL H_2_O and fragmented by sonication (6 × 2 min, 50% intensity, MS72 sonotrode, HD2200 Sonopuls). Fragmented DNA was analyzed on 1% agarose gels to ensure fragment sizes between 200 and 1500 bp. The fragmented DNA was concentrated using Amicon Ultra Centrifugal Filters (30K) (Millipore). Cohesive ends were blunted and blunt ends were phosphorylated according to manufacturer’s instructions (Fast DNA End Repair Kit, Thermo Scientific). DNA was then purified using a spin column according to the manufacturer’s instructions (NucleoSpin Gel and PCR Clean-UP Kit, Macherey-Nagel).

The libraries were constructed by blunt-end cloning of 1,400 ng fragmented metagenomic DNA into 1,000 ng PmeI (NEB) linearized and dephosphorylated (Calf Instestine Phosphatase, NEB) pHORF3^35^ library vector (16 h at 16 °C, T4 DNA Ligase, Promega). The ligation was inactivated for 10 min at 65 °C and the buffer was exchanged 4 times with 500 μL H_2_O using Amicon Ultra Centrifugal Filters (30K) (Millipore). The ligation was splitted into 4 separate transformations and 25 μL of electrocompetent *E. coli* TOP10F’ (TOP10F´ Electrocomp™ Kit, Life Technologies) were transformed by electroporation (1.8 kV, MicroPulser™, BioRad). After 1 h of incubation at 37 °C and 650 rpm in 1 mL SOC medium (2% (w/v) tryptone, 0.5% (w/v) yeast extract, 0.05 (w/v)% NaCl, 10 mM MgCl_2_, 10 mM MgSO_4_, 20 mM glucose), transformation rates were determined by plating dilutions on 2× YT agar (1.6% (w/v) tryptone, 1% (w/v) yeast extract, 0.05% (w/v) NaCl, 1.2% (w/v) agar) supplemented with 100 mM glucose and 100 μg/mL ampicillin (2× YT-GA). Cells were plated on 2× YT-GA agar plates (25 × 25 cm) and incubated at 37 °C overnight. The cells were scraped using 20 mL of 2× YT medium, flash frozen in liquid nitrogen and stored at −80 °C in 20% (v/v) glycerol. Insert rates and mean insert sizes were determined by PCR and capillary gel electrophoresis (Qiaxcel Advance, Qiagen, Germany) of randomly analyzed colonies (n = 20 per transformation).

### Packaging of oligopeptide phage library and ORF enrichment

Four hundred mL of 2× YT-GA medium were inoculated with frozen cells (see above) to an OD_600_ < 0.1 and cultivated at 37 °C and 250 rpm (Infors HT) until an OD_600_ of 0.5 was reached. In order to complement the missing coat proteins and ensure enrichment for ORFs, 25 mL of the culture were infected with a 20-fold excess (2.5 × 10^11^ colony forming units (cfu) of the M13K07ΔgIII helperphage “Hyperphage”^31^) for 30 min at 37 °C. The infected cells were incubated at for another 30 min at 37 °C and 250 rpm (Infors HT) to express antibiotics resistance. The cells were pelleted (3,200× g, 10 min) and subsequently resuspended in 400 mL 2× YT medium supplemented with 100 μg/mL ampicillin and 50 μg/mL kanamycin (2× YT-AK).

Oligopeptide phage particles were produced for 24 h at 30 °C and 250 rpm (Infors HT). The cells were pelleted and phage particles were precipitated from the supernatant at 4 °C overnight after adding 1/5 volume precipitation buffer (20% (w/v) PEG 6,000, 2.5 M NaCl). The precipitated phage particles were pelleted for 1 h at 12,000× g and 4 °C (Sorvall Centrifuge RC5B Plus, Rotor F9S) and resuspended in 10 mL phage dilution buffer (10 mM Tris-HCl pH7.5, 20 mM NaCl, 2 mM EDTA). In order to confer higher purity, the resuspended phage were filtered (Whatman syringe filter, 0.45 μm) and a second precipitation step was performed for 2 h at 4 °C with 1/5 volume precipitation buffer. The phage particles were pelleted for 30 min at 20,000× g and 4 °C (Sorvall Centrifuge RC5B Plus, Rotor SS34) and resupended in 1 mL fresh phage dilution buffer. Remaining bacteria were pelleted for 2 min at 16100× g (Eppendorf Centrifuge 5415 D) and supernatants containing the oligopeptide phage libraries were collected and stored at 4 °C.

### Library panning (selection of candidate immunogenic oligopeptides from complex libraries)

#### Immobilization of serum antibodies

Panning allows the selection of displayed oligopeptides based on specific affinity binding to serum antibodies. In order to reduce background signals, the serum used for panning was precleared from antibodies reactive with phage particles alone. Therefore, two wells of a 96-well ELISA plate (Costar) were coated with 4 × 10^10^ cfu Hyperphage in 300 μL phosphate buffer saline (PBS). The protein binding capacity of the well surface was saturated with 350 μL blocking solution (PBS supplemented with 2% skim milk powder (Roth) and 0.1% Tween 20) per well. The blocking solution was removed and the mouse sera (1:500 dilution in 300 μL blocking buffer) were incubated for 1 h in each of the two wells (total 2 h) with immobilized “Hyperphage” to preclear anti-phage serum antibodies.

The precleared serum was transferred into 2 wells (150 μL each) previously coated with anti-mouse (IgA, IgG and IgM specific) antibodies (antikoerper-online, ABIN376851) and saturated with blocking buffer in order to capture the serum antibodies used for the panning procedure (2 h at room temperature). Excess serum antibodies and other serum proteins were removed by washing 3 times with washing buffer (PBS supplemented with 0.05% Tween 20).

#### Selection of specifically bound oligopeptides

Panning is an enrichment process of specifically reactive oligopeptide phage clones over several consecutive rounds (a schematic illustration is provided in Figure S1). Therefore, three rounds of panning were performed using 2 × 10^10^ cfu oligopeptide phage library (in 150 μL blocking buffer) as input in round one and 3 × 10^11^ cfu of amplified oligopeptide phage as input in rounds two and three.

For the selection of specifically bound oligopeptides, the oligopeptide phage were incubated with the immobilized serum antibodies for 2 h at room temperature. Unbound oligopeptide phage were removed by stringent washing (10 times with washing buffer). Bound oligopeptide phage particles were eluted with 200 μL elution buffer (10 μg/mL trypsin in PBS) per well for 30 min at 37 °C. The eluted phage titer was determined using 10-fold dilutions of 10 μL of the elution followed by *E. coli* TOP10F’ infection and plating onto 2× YT agar supplemented with ampicillin.

#### Amplification of eluted phage

In order to amplify the eluted phage for input in the next panning round, the remaining 390 μL of elutions (see above) were used to infect 5 mL of an *E. coli* TOP10F’ culture (OD_600_ = 0.5) for 30 min at 37 °C. Cells were plated on 15 cm 2× YT-GA agar plates and incubated at 37 °C overnight in order to allow amplification of eluted phage for the next panning round. The cells were scraped using 5 mL of 2× YT-GA. For the production of phage, 30 mL of 2× YT-GA were inoculated (OD_600_ < 0.1) with the scraped cells and cultivated up to OD_600_ = 0.5. Phage particles were produced following the same procedure as described above for packaging of oligopeptide phage libraries. Amplified phage were precipitated only once (1 h on ice) after adding 1/5 volume precipitation buffer. After pelleting (1 h at 4 °C, 3,220× g), the oligopeptide phage were resuspended in 500 μL phage dilution buffer. Remaining bacteria were pelleted for 2 min at 16,100× g, the amplified oligopeptide phage containing supernatant was collected and stored at 4 °C until used as input phage for the next panning round.

After the third panning round, 10-fold dilutions of eluted phage were used to infect 50 μL *E. coli* TOP10F’ (OD_600_ = 0.5), the cells were plated on 2× YT-GA agar plates and incubated at 37 °C overnight in order to obtain single colonies that allow screening of monoclonal oligopeptide phage. To screen the enriched clones after the third round of each panning for immunogenic oligopeptides, 92 randomly selected clones were analyzed by monoclonal phage ELISA.

#### Monoclonal screening ELISA

In order to screen the clones selected in high-throughput manner using TNF^ΔARE/+^ serum antibodies as described above, monoclonal oligopeptide phage were produced. A 96-well microtiter plate (MTP) was supplemented with 180 μL 2× YT-GA medium, inoculated with single colonies after the third panning round (see above) and incubated (34 °C, 800 rpm, Labnet Vortemp 56) overnight. For phage production, 175 μL 2× YT-GA per well were inoculated with 10 μL of the overnight culture and incubated for 2 h at 37 °C and 800 rpm to reach logarithmic growth. The cells were infected with 5 × 10^9^ cfu Hyperphage (M13K07∆gIII) for 30 min at 37 °C. To ensure antibiotic resistance, the cells were incubated for another 30 min at 37 °C and 800 rpm. In order to change the medium for phage production, the cells were pelleted for 10 min at 3,220× g and resuspended in 180 μL 2× YT-AK followed by phage production at 30 °C and 800 rpm overnight. The cells were pelleted and the phage were precipitated by adding 40 μL precipitation buffer to 150 μL phage containing supernatant.

To capture the oligopeptide phage, rabbit anti-M13 (pVIII specific, 1:5,000 in PBS, PA1-46334, Pierce Biotechnology) was immobilized on an ELISA plate (Costar) at 4 °C overnight. Wells were saturated with blocking buffer for 1 h at room temperature. The plates were washed 3 times with washing buffer before adding the monoclonal phage (40 μL in 100 μL blocking buffer). Phage particles were captured for 2 h at room temperature. To reduce background phage binding, the mouse serum samples (1:500) were precleared in blocking buffer containing 10^10^ cfu/mL Hyperphage for 2 h at room temperature. Remaining monoclonal phage were removed by 3 washing steps with washing buffer and the precleared sera were transferred to the wells containing immobilized oligopeptide phage particles and incubated for 2 h at room temperature. The serum was removed by 3 washing steps with washing buffer and bound serum antibodies were detected using goat anti-mouse (IgA, IgG, IgM) antibodies conjugated with horseradish peroxidase (HRP) (antikoerper-online, ABIN376237, 1:8,000 in blocking buffer) for 1.5 h at room temperature. Excess detection antibody was removed by 3 washing steps and the ELISA was developed with TMB substrate solution. Signals were detected using an ELISA reader (TECAN Sunrise, 450 nm, reference 620 nm).

### Immunoblot of oligopeptide phage

In order to validate the immunogenic character of the displayed oligopeptide, staining of the oligopeptide::pIII fusion protein by TNF^ΔARE/+^ sera and wildtype controls was analyzed by immunoblotting of the selected oligopeptide phage. The oligopeptide phage (4 × 10^8^−6 × 10^9^ cfu) proteins were separated on a 12% polyacrylamide gel and blotted onto PVDF membranes. Membranes were blocked by incubating in blocking solution (PBS supplemented with 2% skim milk powder (Roth, Germany)) at 4 °C overnight. Sera of fifteen 18-week-old TNF^ΔARE/+^ and 19 C57BL/6N wt male mice were pooled. These pools and the reference sera used for selection were each diluted 200-fold in blocking solution containing 1.5 × 10^10^ cfu/mL M13K07 wildtype phage for competition and were incubated at 4 °C overnight. Membranes were stained with the sera, mouse anti-hexahistidine (Qiagen Germany, 34660, 1:500 in blocking buffer) and mouse anti-pIII (MoBiTec, Germany, PSKAN3, 1:1,000 in blocking buffer) controls for 2.5 h at room temperature and excess antibodies were removed by using washing buffer (PBS supplemented with 0.1% Tween 20). Bound serum antibodies were detected with a goat anti-mouse (IgA, IgG, IgM) antibody conjugated with HRP (antikoerper-online, ABIN376237, 1:4,000 in blocking buffer) for 1.5 h at room temperature. Immunoblots were developed using diaminobenzidine substrate.

### ELISA based measurement of antibody response

The ELISA plates (Costar) were coated with 200 ng/well streptavidin (Serva) at 4 °C overnight and the wells were blocked with 1× PBS supplemented with 0.1% Tween 20 and 2% BSA. After three washing steps with washing buffer (H_2_O supplemented with 0.05% Tween 20), 200 ng/well of the three selected peptides produces as synthetic biotinylated peptides (Peps4LS, Heidelberg, Germany) were immobilized for 2 h at room temperature. Excess peptides were removed by three washing steps with washing buffer. The mouse sera were diluted 1:100 in blocking buffer (PBS supplemented with 2% skim milk powder (Roth) and 0.1% Tween 20) and incubated with the immobilized peptides and the streptavidin controls for 2.5 h at room temperature. Unbound serum antibodies were removed by three washing steps with washing buffer. The bound serum antibodies were detected with goat anti-mouse (IgA, IgG, IgM) antibodies conjugated with HRP (antikoerper-online, ABIN376237, 1:8,000 in blocking buffer) for 1.5 h at room temperature and the ELISA was developed with TMB substrate solution as described above. Serum reactivity with the peptides was measured in doublets. The measured background binding to streptavidin was subtracted from the mean serum reactivity to compensate inter-individual differences in background binding.

### High-throughput sequencing

For 16S rRNA amplicon analysis, frozen caecal samples were processed, sequenced, and analyzed as described previously^65^. Briefly, DNA was purified after mechanical lysis of microbial cells using NucleoSpin gDNA columns (Macherey-Nagel), the V3-V4 region of 16S rRNA genes was amplified (25 cycles) using primers 341F-785R^66^, and purified using magnetic beads (Beckmann). Sequences obtained by paired-end sequencing using a MiSeq Illumina sequencer were analyzed by in-house developed pipelines following the UPARSE approach^67^ available via the IMNGS platform (www.imngs.org)^68^. Defaults setting and a minimum/maximum length of 400/450 nt for merged reads were used. Operational taxonomic units (OTUs) were picked at a threshold of 97% sequence similarity, and only those occurring at a relative sequence abundance of at least 0.5% in one sample were kept for further analysis.

For next generation sequence analysis of ORF enriched phage libraries, each library was packaged (see above) in triplicates and phagemid preparations from *E. coli* cells after infection with the library oligopeptide phage were used as template. In order to prevent any infection bias, the displayed oligopeptides were removed from the phage particles by treatment with 10 μg/mL trypsin for 30 min at 37 °C (the oligopeptide::pIII fusion protein contains a trypsin site between oligopeptide and pIII). Twenty mL of an *E. coli* TOP10F’ culture (OD_600_ = 0.5) were infected with 1 − 6 × 10^9^ cfu (at least 20-fold excess of inital library diversity) of the oligopeptide phage library (30 min, 37 °C). For plasmid preparation, the cells were plated on 2xYT-GA agar plates, cultured overnight at 37 °C and scraped the next day. The cells were pelleted (10 min, 3,220× g) and plasmid DNA was isolated by NucleoSpin Plasmid EasyPure Kit (Macherey-Nagel, Berlin, Germany) according to manufacturers’ instruction.

Nucleotides libraries were constructed by PCR (25 cycles) using primer Fwd-5′-GCTCAGCCGGCGATGG and Rev-5′-CAGCTCTGATATCTTTGGATCCC. They were purified and sequenced in paired-modus using a MiSeq Illumina sequencer. Reads were merged, cleaned from primer and vector sequences, and those with > 0.1% expected error rate were discarded using USEARCH v8.1.18^69^. When different samples or replicates were compared, sequences were first dereplicated to allow comparison of unique reads across samples.

In order to analyze the insert size distribution and to estimate the capacity for successful translation to oligopeptides of the metagenomic fragments, the sequence reads were trimmed from adapter and vector sequences and the stop codon content of the inserts was analyzed for one of the libraries. Around 4% of those reads had truncated or expanded vector sequences that would modify the correct reading frame in the analysis and were removed. All inserts were scanned (starting from the 5′ end and translating downstream) for stop codons (TAA, TGA or TAG) that would interrupt the translation. The number of sequences which could be rescued by alternative start codons (ATG) downstream of the last stop codon were determined by walking in opposite direction (from 3′ end) to ensure alternative start codons have the potential to initiate translation of in-frame products.

### Statistical analysis

Statistical analysis was performed by non-parametric Mann-Whitney test using the GraphPad Prism software package.

## Additional Information

**How to cite this article**: Zantow, J. *et al*. Mining gut microbiome oligopeptides by functional metaproteome display. *Sci. Rep.* **6**, 34337; doi: 10.1038/srep34337 (2016).

## Supplementary Material

Supplementary Information

## Figures and Tables

**Table 1:** Summary of selected clones.

Clone	Origin mouse	Insertsize (bp)	Hit redundancy	Protein homology (blastp)
JOZ156-A3	1	43	1	—
JOZ156-A6	1	298	32	Predicted protein (*Blautia* sp.) (E-value 7e-24, 66 % identity)
JOZ156-A8	1	319	1	Predicted protein (*Blautia* sp.) (E-value 3e-27, 57 % identity)
JOZ156-B11	1	58	6	—
JOZ156-F7	1	328	1	Predicted protein (*Blautia* sp.) (E-value 3e-29, 58 % identity)
JOZ156-G7	1	85	2	—
JOZ156-H5	1	58	1	—
JOZ157-C10	2	211	3	Predicted protein (*Blautia* sp.) (E-value 7e-18, 72 % identity)
JOZ157-E1	2	202	1	ABC transporter permease (*Eubacterium* sp. ER2) (E-value 2e-21, 62 % identity)
JOZ157-G6	2	58	1	—
JOZ158-C11	3	76	1	—
JOZ158-C12	3	46	1	—
JOZ158-D10	3	31	2	—
JOZ158-E11	3	121	2	Phosphoribosylaminoimidazol-succinocarboxamide synthase (*Blautia* sp.) (E-value 4e-20, 100 % identity)
JOZ158-G8	3	163	1	—
JOZ158-H8	3	52	1	—
