# Supplementary material for: Mining gut microbiome oligopeptides by functional metaproteome display
Source: Sci Rep. 2016 Oct 5;6:34337. doi: 10.1038/srep34337 (PMC5050496; doi:10.1038/srep34337)
Supplement: Supplementary Information [file srep34337-s1.pdf]

Supplementary Information

Mining gut microbiome oligopeptides by functional metaproteome display

Jonas Zantow[1], Sarah Just[2], Ilias Lagkouvardos[2], Sigrid Kisling[2], Stefan Dübel[1], Patricia Lepage[3], Thomas Clavel[2,*] and Michael Hust[1,*]

[1]Technische Universität Braunschweig, Institute of Biochemistry, Biotechnology and Bioinformatics – Department for Biotechnology, Germany

[2]Technische Universität München, ZIEL Institute for Food and Health, Freising, Germany

[3]Micalis Institute, INRA, AgroParisTech, Université Paris-Saclay, Jouy-en-Josas, France

*Shared senior and corresponding authorship

Figure legends

Figure S1: Schematic illustration of the ORFeome phage display technology. A) Construction of metaproteome libraries B) ORF enrichment and functional display C) oligopeptide selection with immobilized serum antibodies

Figure S2: Size distribution histogram of the metaproteome libraries. Sequences with 3n, 3n+1 and 3n+2 nucleotides are displayed in black, light grey, and dark grey. Enriched sequences with 3n+1 nucleotides represent an ORF enrichment in the libraries. The histogram is limited to sequences from 0 to 300 nucleotides (nt) (total sequence range was 8 to 476 nt). Analysis of 107,330 unique reads (size detection limit in NGS was approx. 500 nucleotides).

Figure S3: Illustration of the cloning site in the ORFeome phage display vector pHORF3. Only inserts with 3n+1 nucleotides result in open reading frames with pelB leader sequence, hexahistidine tag and M13 minor coat protein III gene (gIII).

Figure S4: Enrichment of binding oligopeptide phage over the panning rounds. Phage titers were determined by infecting *E. coli* with dilution series of phage and spotting on ampicillin supplemented medium.

Suppl. Tables

Table S1: Stop codon analysis of ORF-enriched metaproteome phage library

Insert type (nt)	% of total reads	% in insert type				
		stop codon	alternative start codon	stop codon and alternative start codon	stop codon and no alternative start codon	potential pIII gene products
3n+1	70.3	3.4	38.6	0.6	2.8	97.2
3n+2	27.3	2.7	29.9	1.1	1.5	29.9
3n	2.4	43.0	28.4	21.4	21.6	28.4

Table S2: Summary of selected and validated biomarker candidates

Clone	DNA sequence	Peptide Sequence	Homologue protein (blastp)
JOZ156-H5	GACTTCATGGGGAACTTTT<br>GGAATTTTAATTCCTATCG<br>CGACTTCGATTTTCCC	DFMGNFQNFNS<br>YRDFDFP	-
JOZ158-E11	TGGCCTGCTGACGGATAT<br>GAACCGGGACACGGTCAG<br>CCTTCCTTTGACAAGCAGT<br>TTGCCCGCGACTGGCTGA<br>AAGAAAATGACGGTCATGA<br>CTGGACTCTTCCTCAGGA<br>GATCGTT	WPADGYEPGHG<br>QPSFDKQFARD<br>WLKENDGHDWT<br>LPQEIV	Phosphoribosyl-aminoimidazole-succinocarbox-amide synthase (*Blautia* sp. (E-value 4e-20, 100 % identity))
JOZ158-G8	AACGACCTGAACGCGCTG<br>CTTGAGACAAACATATCCG<br>CGGGAACCAGAACTACCC<br>AGGTATCTATGAGCTATTT<br>CGGGGAAATGCTTATGAG<br>CCATATCGCGGACAATGAA<br>TTCTTAAGCGGCTCGGAA<br>GATGAAAAGGCGGCTCAC<br>GCAAATGCGCTT	NDLALLETNISAG<br>TRTTQVSMSYF<br>GEMLMSHIADNE<br>FLSGSEDEKAAH<br>ANAL	-

Suppl. Figures

Figure S1:

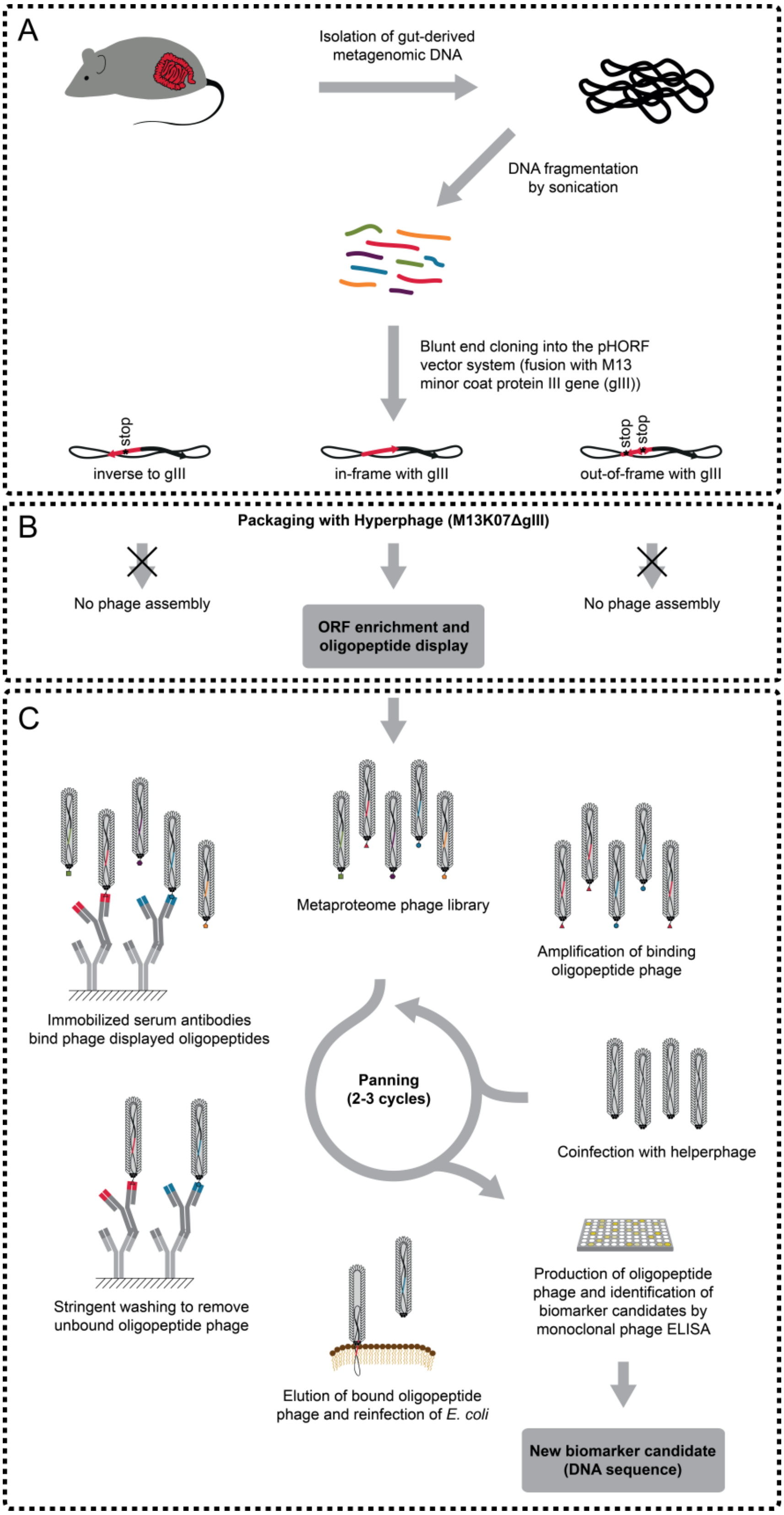

Figure S2:

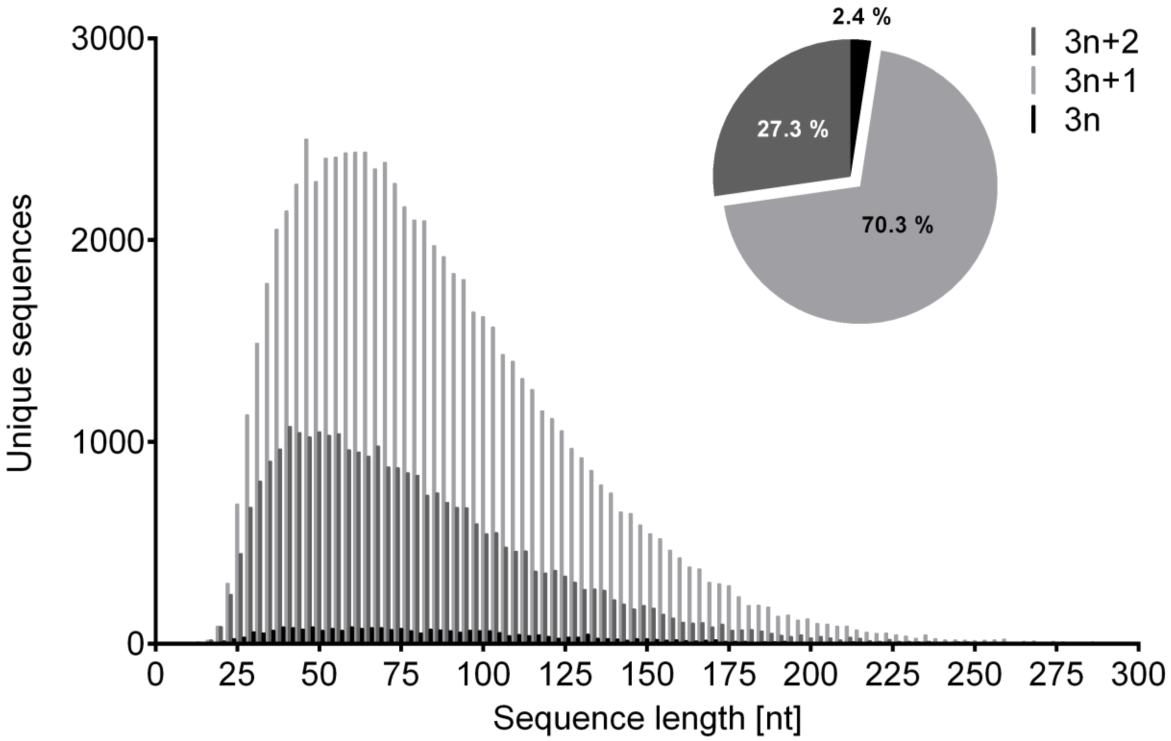
2.4 %
27.3 %
70.3 %
3n+2
3n+1
3n
Unique sequences
0
1000
2000
3000
0
25
50
75
100
125
150
175
200
225
250
275
300
Sequence length [nt]

Figure S3:

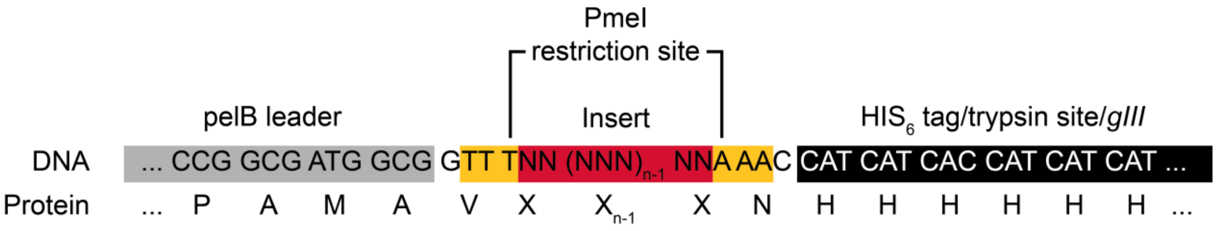

PmeI
restriction site
pelB leader
Insert
HIS6 tag/trypsin site/gIII
DNA
... CCG GCG ATG GCG GTT TNN (NNN)n-1 NNA AAC CAT CAT CAC CAT CAT CAT ...
Protein
... P A M A V X Xn-1 X N H H H H H H ...

Figure S4:

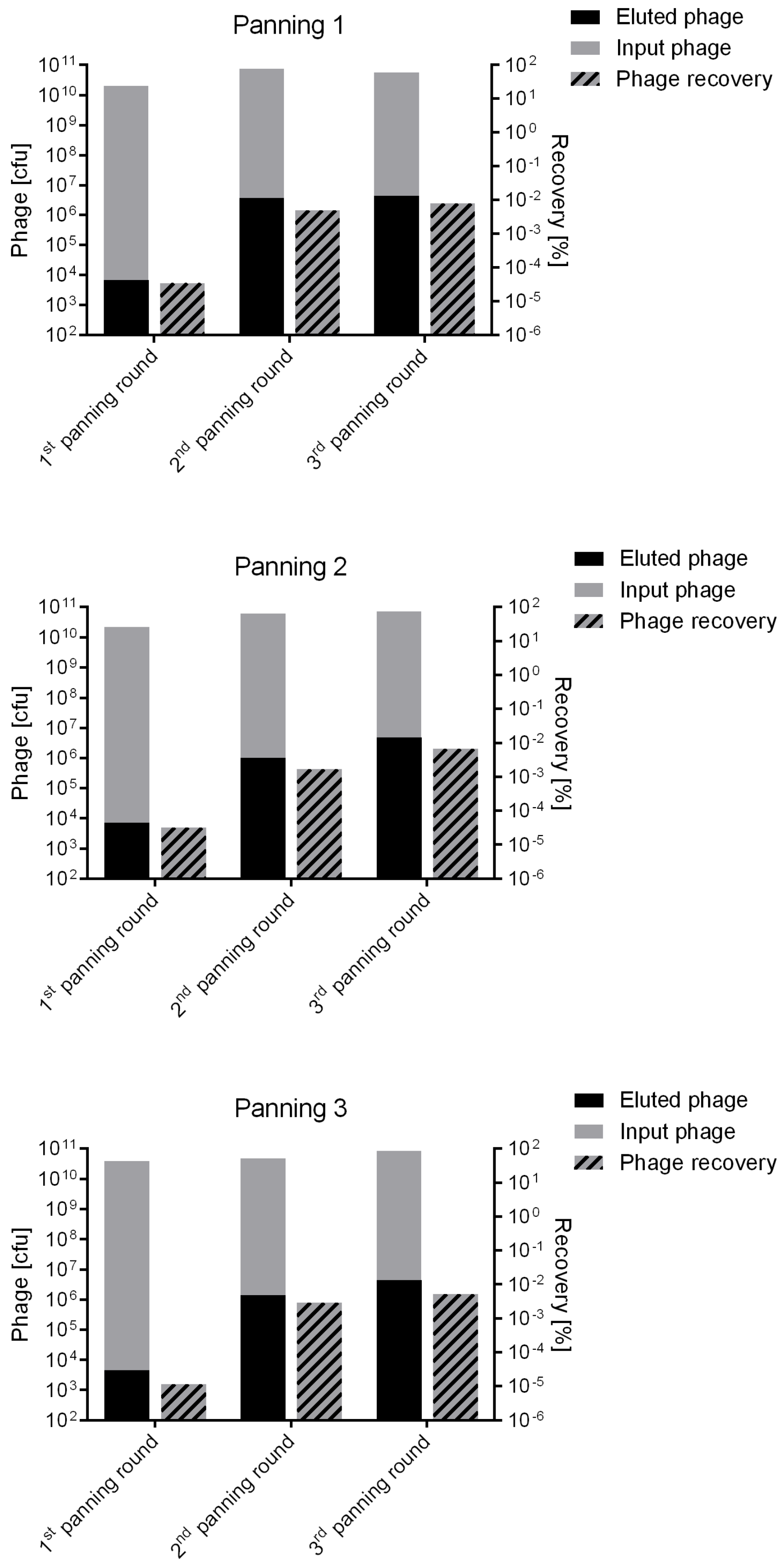
Panning 1
Eluted phage
Input phage
Phage recovery
Phage [cfu]
Recovery [%]
1st panning round
2nd panning round
3rd panning round
Panning 2
Eluted phage
Input phage
Phage recovery
Phage [cfu]
Recovery [%]
1st panning round
2nd panning round
3rd panning round
Panning 3
Eluted phage
Input phage
Phage recovery
Phage [cfu]
Recovery [%]
1st panning round
2nd panning round
3rd panning round
